# Al_48.18_Cr_22.78_Fe_4.04_Si_3_

**DOI:** 10.1107/S2414314625010399

**Published:** 2025-11-21

**Authors:** Xinyu Liang, Changzeng Fan, Bin Wen, Lifeng Zhang

**Affiliations:** a State Key Laboratory of Metastable Materials Science and Technology Yanshan University,Qinhuangdao 066004 People’s Republic of China; b Hebei Key Lab for Optimizing Metal Product Technology and Performance Yanshan University,Qinhuangdao 066004 People’s Republic of China; c School of Mechanical and Materials Engineering North China University of Technology,Beijing 100144 People’s Republic of China; Benemérita Universidad Autónoma de Puebla, México

**Keywords:** crystal structure, high-pressure sinter­ing, inter­metallic, deca­gonal quasicrystal

## Abstract

A mixture with the initial chemical composition Al_59_Cr_21_Fe_10_Si_10_ was subjected to high-pressure sinter­ing, resulting in the unexpected synthesis of an inter­metallic compound with the composition Al_48.18_Cr_22.78_Fe_4.04_Si_3_, which adopts the Cr_0.88_Fe_0.12_Ga structure type in the space group *R*3*m*. Structural analysis indicates that Al, Cr and Fe jointly occupy a special position (Wyckoff site 18*h*), with refined site occupancy factors of 0.676 (6), 0.17 (2) and 0.16 (2), respectively. Cr and Fe share another special position (Wyckoff site 3*b*), with refined site occupancy factors of 0.60 (6) and 0.40 (6), respectively. Other atoms in the crystal (Al and Si) fully occupy their sites.

## Abstract 2

The title quaternary inter­metallic material is a new γ-brass obtained using a high-pressure sinter­ing process.

## Graphical abstract

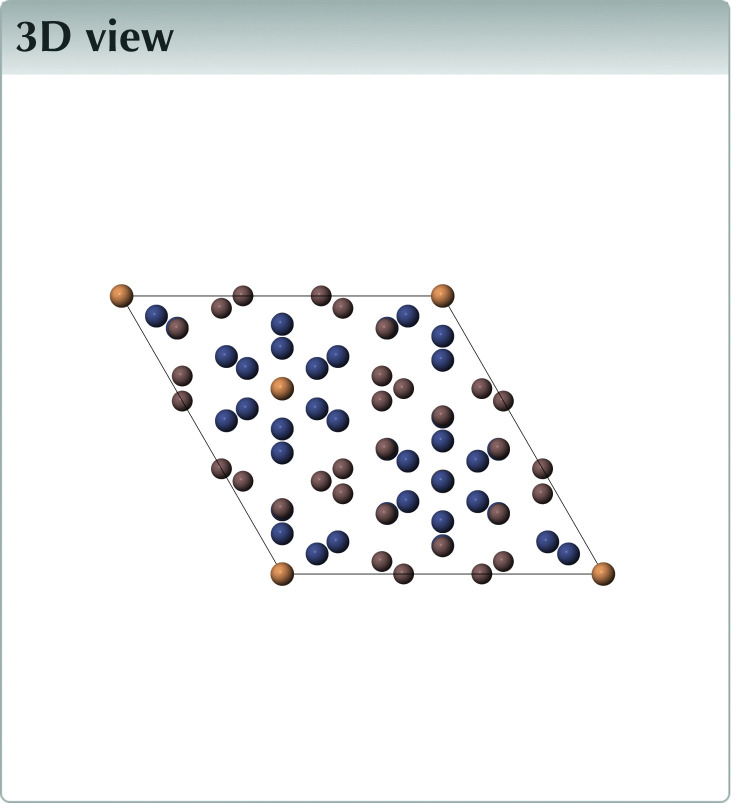

## Structure description

Quaternary deca­gonal quasicrystals (DQC) have been discovered in a few systems, for example, Ga–Fe–Cu–Si, Ga–V–Ni–Si and Al–Mn–Fe–Ge. However, there have been far fewer detailed studies of the stability of quaternary DQCs and correspondingly, there is far less atomic-scale structural information. The quaternary Al–Cr–Fe–Si DQCs exhibit distinctive structural characteristics, thereby constituting a novel structural class of DQCs. Specifically, the presence of multiple types of 2-nm deca­gons exhibiting both perfect and destructive tenfold symmetry within Al–Cr–Fe–Si DQCs is noteworthy, as such a phenomenon is rarely observed in other DQCs. This observation is likely to generate considerable inter­est, as the 2-nm deca­gons constitute the most significant structural element of DQCs. Therefore, it is imperative that an exhaustive study of the thermodynamic stability and the corresponding atomic-scale structural information to be conducted.

Ma *et al.* (2018 ▸, 2020 ▸) discovered the DQC Al_59_Cr_21_Fe_10_Si_10_ by melting the quaternary alloy Al_60_Cr_20_Fe_10_Si_10_ in an induction furnace under an argon atmosphere, using high-purity elements. The molten alloy was then poured into a graphite crucible in the furnace to form ingots. Some fragments of the ingots were sealed in vacuum tubes for heat treatment.

In the present study we synthesized a new γ-brass phase, namely Al_48.18_Cr_22.78_Fe_4.04_Si_3_ utilizing a high-temperature and high-pressure method based on the composition Al_59_Cr_21_Fe_10_Si_10_, as detailed in the *Synthesis* section. It is evident that the system under consideration shares many similarities with other systems with a similar structure. For example, Hu *et al.* (2021 ▸) found Al_8.6_Mn_4.4_ in the same space group [*a* = 12.6751 (13), *c* = 7.9137 (9) Å]; Ko *et al.* (2010 ▸) found Cr_0.88_Fe_0.12_Ga [*a* = *b* = 12.6431 (18), *c* = 7.8985 (16) Å]. The structure of Al_48.18_Cr_22.78_Fe_4.04_Si_3_ is similar to that of Cr_0.88_Fe_0.12_Ga; in the former, Al, Cr and Fe share a special position (Wyckoff position 18*h* in the space group *R*
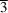
*m*), with refined site occupancy factors of 0.676 (6), 0.17 (2) and 0.16 (2), while in the latter, Fe and Cr share this position, with refined site occupancy factors of 0.26 and 0.74 (Ko *et al.*, 2010 ▸). However, no reports are available so far for the quaternary alloy phase of Al–Cr–Fe–Si with such a structure.

**Figure 1:**
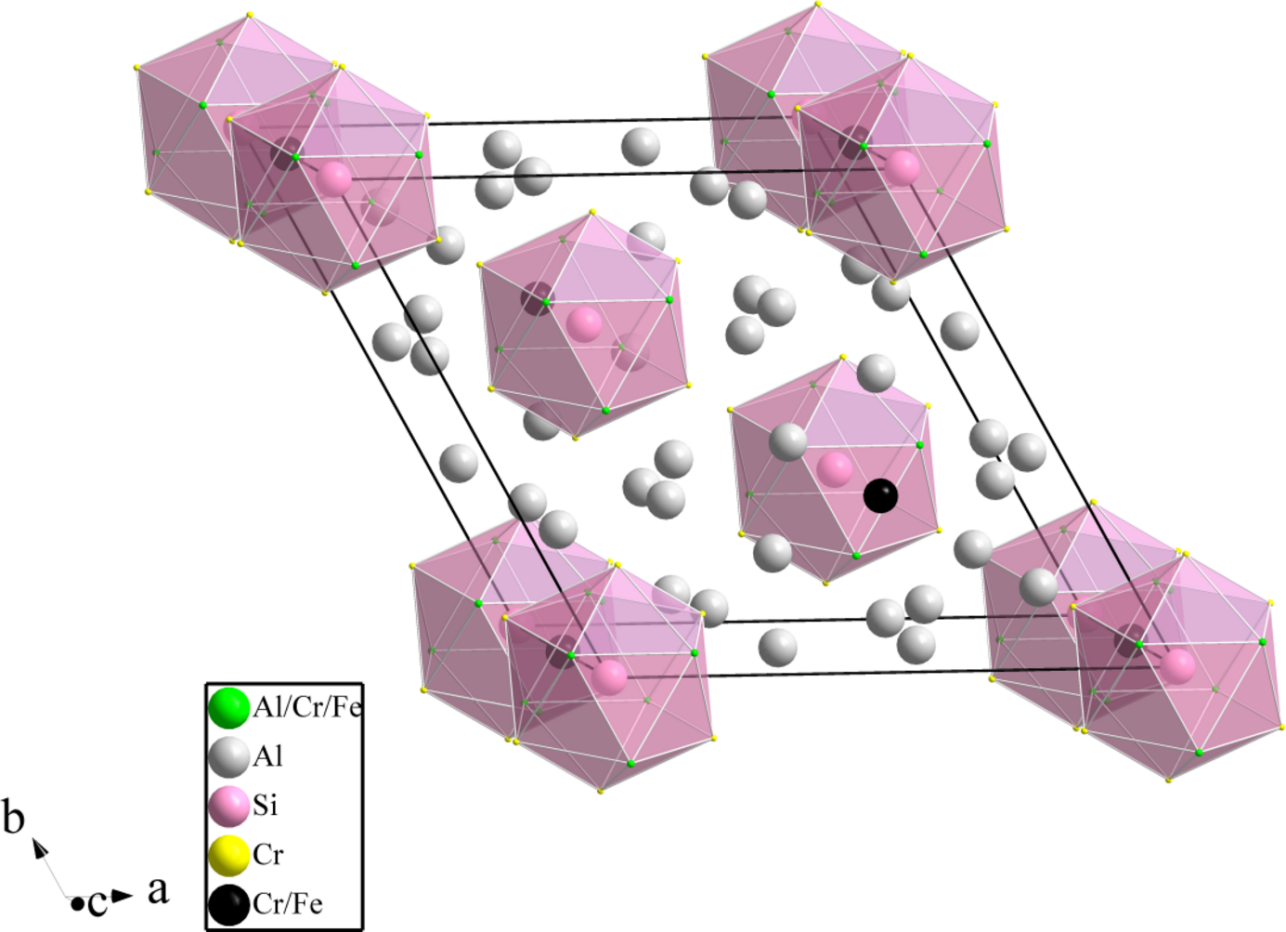
The crystal structure of Al_48.18_Cr_22.78_Fe_4.04_Si_3_. The icosa­hedra centred on Si1 are emphasized.

In this study, we refined the crystal structure model of Al_48.18_Cr_22.78_Fe_4.04_Si_3_ based on single-crystal X-ray diffraction data. Its chemical composition is in accordance with EDX measurements; various attempts are made with different results at each position (see the supporting information). The crystal structure and parts thereof are shown in Figs. 1 ▸ and 2 ▸,

## Synthesis and crystallization

The high-purity elements Al (indicated purity 99.8%; 0.4519 g), Cr (indicated purity 99.95%; 0.3099 g), Fe (indicated purity 99.9%; 0.1585 g), and Si (indicated purity 99.9%; 0.0797 g) were uniformly mixed in a stoichiometric ratio of 59:21:10:10 and thoroughly ground in an agate mortar. The mixed powder was placed into a cemented carbide grinding mould of 5 mm diameter and pressed into a block under a pressure of approximately 5 MPa for 3 min. A cylindrical block was obtained without deformations or cracks. The experimental details of high-pressure sinter­ing using a six-anvil high-temperature high-pressure device can be consulted in Liu & Fan (2018 ▸).

In the current work, the prepared cylindrical block mixture was pressurized up to 6 GPa and heated to 1473 K for 30 min., cooled to 1173 K, held at that temperature for 60 min., and then rapidly cooled down to room temperature. A fragment was selected and mounted on a glass fibre for single-crystal X-ray diffraction measurements.

## Refinement

Table 1 ▸ shows the details of data collection and structural refinement. The labelling scheme and atomic coordinates of Al_48.18_Cr_22.78_Fe_4.04_Si_3_ were adapted from Cr_0.88_Fe_0.12_Ga for better comparison. One site is co-occupied by Al, Cr and Fe atoms (Al1/Cr3/Fe1), and site occupancies were refined to 0.676 (6) for Al, 0.17 (2) for Cr and 0.16 (2) for Fe. Another site is co-occupied by Cr and Fe (Cr2/Fe2), and site occupancies were refined to 0.60 (6) for Cr and 0.40 (6) for Fe. Atoms sharing the same position were constrained to have identical coordinates and displacement parameters. The maximum and minimum residual electron densities in the last difference map are located 1.18 Å from atom Si1 and 0.75 Å from atom Al3, respectively.

## Supplementary Material

Crystal structure: contains datablock(s) I. DOI: 10.1107/S2414314625010399/bh4100sup1.cif

Structure factors: contains datablock(s) I. DOI: 10.1107/S2414314625010399/bh4100Isup4.hkl

Figures and supplementary materials. DOI: 10.1107/S2414314625010399/bh4100sup3.zip

CCDC reference: 2504093

Additional supporting information:  crystallographic information; 3D view; checkCIF report

## Figures and Tables

**Figure 2:**
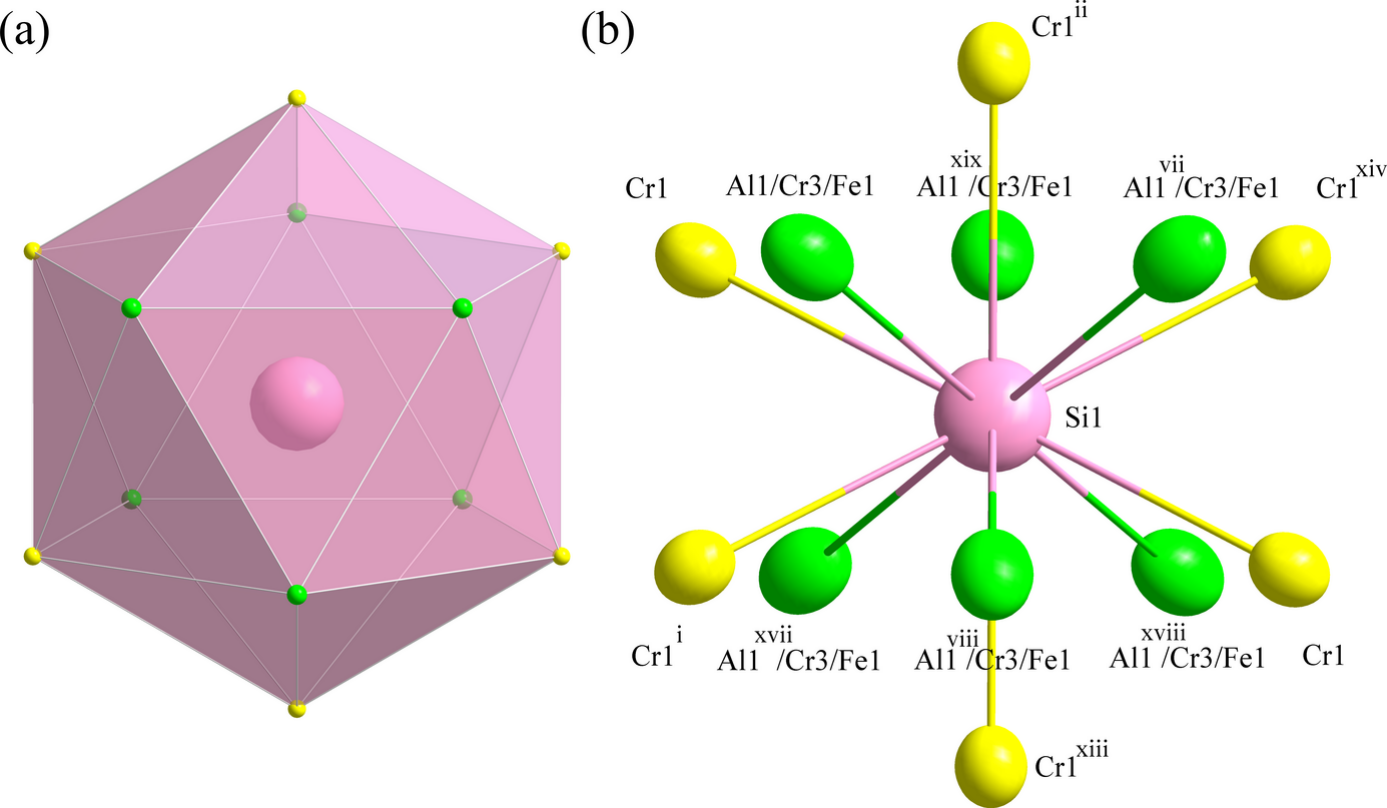
(*a*) The icosa­hedra formed around the Si1 atom at the 3*a* site; (*b*) the environment of the Si1 atom with displacement ellipsoids given at the 99% probability level. [Symmetry codes: (i) *x* − 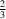, *y* − 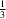, *z* − 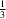; (ii) −*x* + *y* + 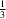, −*x* + 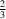, *z* − 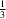; (vii) −*x* + *y*, −*x*, *z*; (viii) −*y*, *x* − *y*, *z*; (xiii) *x* − *y* − 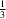, *x* − 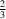, −*z* + 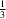; (xiv) −*x* + 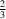, −*y* + 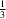, −*z* + 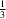; (xvii) *x* − *y*, *x*, −*z*; (xviii) −*x*, −*y*, −*z*; (xix) *y*, −*x* + *y*, −*z*.]

**Table 1:** Experimental details

Crystal data	
Chemical formula	Al_48.18_Cr_22.78_Fe_4.04_Si_3_
*M*_r_	2794.43
Crystal system, space group	Trigonal, *R* 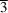 *m*:*H*
Temperature (K)	296
*a*, *c* (Å)	12.5478 (9), 7.9296 (9)
*V* (Å^3^)	1081.2 (2)
*Z*	1
Radiation type	Mo *K*α
μ (mm^−1^)	7.85
Crystal size (mm)	0.08 × 0.06 × 0.06

Data collection	
Diffractometer	Bruker D8 Venture Photon 100 CMOS
Absorption correction	Multi-scan (*SADABS*; Krause *et al.*, 2015 ▸)
*T*_min_, *T*_max_	0.574, 0.746
No. of measured, independent and observed [*I* > 2σ(*I*)] reflections	10693, 417, 338
*R*_int_	0.099
(sin θ/λ)_max_ (Å^−1^)	0.715

Refinement	
*R*[*F*^2^ > 2σ(*F*^2^)], *wR*(*F*^2^), *S*	0.031, 0.047, 1.13
No. of reflections	417
No. of parameters	32
No. of restraints	1
Δρ_max_, Δρ_min_ (e Å^−3^)	0.55, −1.04

Computer programs: *APEX5* and *SAINT* (Bruker, 2023 ▸), *SHELXT2018/2* (Sheldrick, 2015*a* ▸), *SHELXL2018/3* (Sheldrick, 2015*b* ▸), *DIAMOND* (Brandenburg & Putz, 2017 ▸) and *publCIF* (Westrip, 2010 ▸).

## full crystallographic data

### Aluminium chromium iron silicide Crystal data

**Table:**

Al_48.18_Cr_22.78_Fe_4.04_Si_3_	*D*_x_ = 4.292 Mg m^−^^3^
*M**_r_* = 2794.43	Mo *K*α radiation, λ = 0.71073 Å
Trigonal, *R*3*m*:*H*	Cell parameters from 2885 reflections
*a* = 12.5478 (9) Å	θ = 3.2–28.5°
*c* = 7.9296 (9) Å	µ = 7.85 mm^−^^1^
*V* = 1081.2 (2) Å^3^	*T* = 296 K
*Z* = 1	Lump, grey
*F*(000) = 1320	0.08 × 0.06 × 0.06 mm

### Aluminium chromium iron silicide Data collection

**Table:**

Bruker D8 Venture Photon 100 CMOS diffractometer	338 reflections with *I* > 2σ(*I*)
phi and ω scans	*R*_int_ = 0.099
Absorption correction: multi-scan (SADABS; Krause *et al.*, 2015)	θ_max_ = 30.5°, θ_min_ = 3.2°
*T*_min_ = 0.574, *T*_max_ = 0.746	*h* = −17→17
10693 measured reflections	*k* = −17→17
417 independent reflections	*l* = −11→11

### Aluminium chromium iron silicide Refinement

**Table:**

Refinement on *F*^2^	1 restraint
Least-squares matrix: full	Primary atom site location: dual
*R*[*F*^2^ > 2σ(*F*^2^)] = 0.031	Secondary atom site location: difference Fourier map
*wR*(*F*^2^) = 0.047	*w* = 1/[σ^2^(*F*_o_^2^) + (0.0136*P*)^2^ + 4.4686*P*] where *P* = (*F*_o_^2^ + 2*F*_c_^2^)/3
*S* = 1.13	(Δ/σ)_max_ = 0.004
417 reflections	Δρ_max_ = 0.55 e Å^−^^3^
32 parameters	Δρ_min_ = −1.03 e Å^−^^3^

### Aluminium chromium iron silicide Fractional atomic coordinates and isotropic or equivalent isotropic displacement parameters (Å^2^)

**Table:**

	*x*	*y*	*z*	*U*_iso_*/*U*_eq_	Occ. (<1)
Al1	−0.07243 (4)	0.07243 (4)	0.24743 (10)	0.0082 (3)	0.676 (6)
Al2	0.37822 (10)	0.37822 (10)	0.500000	0.0096 (3)	
Al3	0.23539 (11)	0.11769 (5)	0.57349 (15)	0.0108 (3)	
Cr3	−0.07243 (4)	0.07243 (4)	0.24743 (10)	0.0082 (3)	0.17 (2)
Si1	0.000000	0.000000	0.000000	0.0127 (6)	
Fe1	−0.07243 (4)	0.07243 (4)	0.24743 (10)	0.0082 (3)	0.16 (2)
Cr1	0.43537 (5)	0.21768 (3)	0.40484 (7)	0.00680 (18)	
Cr2	0.000000	0.000000	0.500000	0.0035 (4)	0.60 (6)
Fe2	0.000000	0.000000	0.500000	0.0035 (4)	0.40 (6)

### Aluminium chromium iron silicide Atomic displacement parameters (Å^2^)

**Table:**

	*U*^11^	*U*^22^	*U*^33^	*U*^12^	*U*^13^	*U*^23^
Al1	0.0089 (4)	0.0089 (4)	0.0056 (4)	0.0033 (4)	−0.00109 (17)	0.00109 (17)
Al2	0.0107 (5)	0.0107 (5)	0.0092 (5)	0.0068 (5)	0.0011 (2)	−0.0011 (2)
Al3	0.0066 (6)	0.0125 (5)	0.0114 (6)	0.0033 (3)	−0.0012 (5)	−0.0006 (2)
Cr3	0.0089 (4)	0.0089 (4)	0.0056 (4)	0.0033 (4)	−0.00109 (17)	0.00109 (17)
Si1	0.0162 (9)	0.0162 (9)	0.0057 (12)	0.0081 (5)	0.000	0.000
Fe1	0.0089 (4)	0.0089 (4)	0.0056 (4)	0.0033 (4)	−0.00109 (17)	0.00109 (17)
Cr1	0.0084 (3)	0.0068 (3)	0.0057 (3)	0.00420 (17)	0.0003 (2)	0.00016 (12)
Cr2	0.0035 (5)	0.0035 (5)	0.0036 (7)	0.0017 (3)	0.000	0.000
Fe2	0.0035 (5)	0.0035 (5)	0.0036 (7)	0.0017 (3)	0.000	0.000

### Aluminium chromium iron silicide Geometric parameters (Å, º)

**Table:**

Al1—Si1	2.5154 (9)	Al2—Al2^ix^	2.8175 (8)
Al1—Fe2	2.5473 (8)	Al2—Al3	2.8947 (12)
Al1—Cr2	2.5473 (8)	Al2—Al3^iv^	2.8948 (12)
Al1—Cr1^i^	2.6048 (7)	Al3—Cr1	2.5516 (13)
Al1—Cr1^ii^	2.6048 (7)	Al3—Al3^xi^	2.591 (2)
Al1—Al2^i^	2.6344 (10)	Al3—Fe2	2.6234 (12)
Al1—Al2^iii^	2.6344 (10)	Al3—Cr2	2.6234 (12)
Al1—Al3^iv^	2.6478 (11)	Al3—Al3^iv^	2.8108 (14)
Al1—Al3^v^	2.6478 (11)	Al3—Al3^xii^	2.8108 (14)
Al1—Cr1^vi^	2.6979 (10)	Al3—Cr1^xi^	2.8154 (14)
Al1—Al1^vii^	2.7264 (16)	Si1—Cr1^xiii^	2.5766 (6)
Al1—Al1^viii^	2.7264 (16)	Si1—Cr1^ii^	2.5766 (6)
Al2—Cr1^iv^	2.5663 (4)	Si1—Cr1^i^	2.5766 (6)
Al2—Cr1	2.5663 (4)	Si1—Cr1^xiv^	2.5766 (6)
Al2—Cr1^ix^	2.6705 (8)	Cr1—Cr1^x^	2.7575 (7)
Al2—Cr1^x^	2.6705 (8)	Cr1—Cr1^xv^	2.7576 (7)
Al2—Al2^ii^	2.8175 (8)		
			
Si1—Al1—Fe2	103.10 (3)	Al1^xix^—Si1—Cr1^xiii^	116.03 (2)
Si1—Al1—Cr2	103.10 (3)	Al1^viii^—Si1—Cr1^xiii^	63.97 (2)
Si1—Al1—Cr1^i^	60.40 (2)	Al1—Si1—Cr1^xiii^	118.481 (11)
Cr2—Al1—Cr1^i^	110.23 (2)	Al1^xvii^—Si1—Cr1^ii^	118.482 (11)
Si1—Al1—Cr1^ii^	60.40 (2)	Al1^vii^—Si1—Cr1^ii^	61.518 (11)
Fe2—Al1—Cr1^ii^	110.23 (2)	Al1^xviii^—Si1—Cr1^ii^	118.481 (11)
Cr2—Al1—Cr1^ii^	110.23 (2)	Al1^xix^—Si1—Cr1^ii^	63.97 (2)
Cr1^i^—Al1—Cr1^ii^	113.37 (4)	Al1^viii^—Si1—Cr1^ii^	116.03 (2)
Si1—Al1—Al2^i^	107.27 (3)	Al1—Si1—Cr1^ii^	61.519 (11)
Cr2—Al1—Al2^i^	132.19 (3)	Cr1^xiii^—Si1—Cr1^ii^	180.0
Cr1^i^—Al1—Al2^i^	58.65 (2)	Al1^xvii^—Si1—Cr1^i^	63.97 (2)
Cr1^ii^—Al1—Al2^i^	116.70 (4)	Al1^vii^—Si1—Cr1^i^	116.03 (2)
Si1—Al1—Al2^iii^	107.27 (3)	Al1^xviii^—Si1—Cr1^i^	118.481 (11)
Cr2—Al1—Al2^iii^	132.19 (3)	Al1^xix^—Si1—Cr1^i^	118.482 (11)
Cr1^i^—Al1—Al2^iii^	116.70 (4)	Al1^viii^—Si1—Cr1^i^	61.518 (11)
Cr1^ii^—Al1—Al2^iii^	58.65 (2)	Al1—Si1—Cr1^i^	61.519 (11)
Al2^i^—Al1—Al2^iii^	70.91 (5)	Cr1^xiii^—Si1—Cr1^i^	64.698 (9)
Si1—Al1—Al3^iv^	110.40 (3)	Cr1^ii^—Si1—Cr1^i^	115.302 (9)
Cr2—Al1—Al3^iv^	60.62 (3)	Al1^xvii^—Si1—Cr1^xiv^	116.03 (2)
Cr1^i^—Al1—Al3^iv^	166.60 (4)	Al1^vii^—Si1—Cr1^xiv^	63.97 (2)
Cr1^ii^—Al1—Al3^iv^	64.82 (3)	Al1^xviii^—Si1—Cr1^xiv^	61.519 (11)
Al2^i^—Al1—Al3^iv^	134.61 (4)	Al1^xix^—Si1—Cr1^xiv^	61.518 (11)
Al2^iii^—Al1—Al3^iv^	74.44 (3)	Al1^viii^—Si1—Cr1^xiv^	118.482 (11)
Si1—Al1—Al3^v^	110.40 (3)	Al1—Si1—Cr1^xiv^	118.481 (11)
Cr2—Al1—Al3^v^	60.62 (3)	Cr1^xiii^—Si1—Cr1^xiv^	115.302 (9)
Cr1^i^—Al1—Al3^v^	64.82 (3)	Cr1^ii^—Si1—Cr1^xiv^	64.698 (9)
Cr1^ii^—Al1—Al3^v^	166.60 (4)	Cr1^i^—Si1—Cr1^xiv^	180.000 (16)
Al2^i^—Al1—Al3^v^	74.44 (3)	Al3—Cr1—Al2^xii^	68.89 (3)
Al2^iii^—Al1—Al3^v^	134.61 (4)	Al3—Cr1—Al2	68.89 (3)
Al3^iv^—Al1—Al3^v^	113.57 (6)	Al2^xii^—Cr1—Al2	135.23 (5)
Si1—Al1—Cr1^vi^	59.12 (2)	Al3—Cr1—Si1^xvi^	161.11 (3)
Cr2—Al1—Cr1^vi^	162.21 (4)	Al2^xii^—Cr1—Si1^xvi^	107.50 (3)
Cr1^i^—Al1—Cr1^vi^	62.639 (19)	Al2—Cr1—Si1^xvi^	107.50 (3)
Cr1^ii^—Al1—Cr1^vi^	62.639 (19)	Al3—Cr1—Al1^ix^	106.49 (3)
Al2^i^—Al1—Cr1^vi^	60.09 (2)	Al2^xii^—Cr1—Al1^ix^	61.25 (3)
Al2^iii^—Al1—Cr1^vi^	60.09 (2)	Al2—Cr1—Al1^ix^	119.13 (3)
Al3^iv^—Al1—Cr1^vi^	122.79 (3)	Si1^xvi^—Cr1—Al1^ix^	58.08 (2)
Al3^v^—Al1—Cr1^vi^	122.79 (3)	Al3—Cr1—Al1^xvi^	106.49 (3)
Si1—Al1—Al1^vii^	57.184 (16)	Al2^xii^—Cr1—Al1^xvi^	119.13 (3)
Fe2—Al1—Al1^vii^	57.646 (16)	Al2—Cr1—Al1^xvi^	61.25 (3)
Cr2—Al1—Al1^vii^	57.646 (16)	Si1^xvi^—Cr1—Al1^xvi^	58.08 (2)
Cr1^i^—Al1—Al1^vii^	108.20 (2)	Al1^ix^—Cr1—Al1^xvi^	63.11 (4)
Cr1^ii^—Al1—Al1^vii^	58.44 (2)	Al3—Cr1—Al2^ii^	90.98 (3)
Al2^i^—Al1—Al1^vii^	164.434 (19)	Al2^xii^—Cr1—Al2^ii^	129.54 (2)
Al2^iii^—Al1—Al1^vii^	112.54 (2)	Al2—Cr1—Al2^ii^	65.06 (3)
Al3^iv^—Al1—Al1^vii^	59.01 (2)	Si1^xvi^—Cr1—Al2^ii^	104.440 (18)
Al3^v^—Al1—Al1^vii^	108.77 (3)	Al1^ix^—Cr1—Al2^ii^	162.44 (3)
Cr1^vi^—Al1—Al1^vii^	107.55 (2)	Al1^xvi^—Cr1—Al2^ii^	110.76 (3)
Si1—Al1—Al1^viii^	57.184 (16)	Al3—Cr1—Al2^xv^	90.98 (3)
Cr2—Al1—Al1^viii^	57.646 (16)	Al2^xii^—Cr1—Al2^xv^	65.06 (3)
Cr1^i^—Al1—Al1^viii^	58.44 (2)	Al2—Cr1—Al2^xv^	129.54 (2)
Cr1^ii^—Al1—Al1^viii^	108.20 (2)	Si1^xvi^—Cr1—Al2^xv^	104.441 (18)
Al2^i^—Al1—Al1^viii^	112.54 (2)	Al1^ix^—Cr1—Al2^xv^	110.76 (3)
Al2^iii^—Al1—Al1^viii^	164.434 (19)	Al1^xvi^—Cr1—Al2^xv^	162.44 (3)
Al3^iv^—Al1—Al1^viii^	108.77 (3)	Al2^ii^—Cr1—Al2^xv^	69.81 (5)
Al3^v^—Al1—Al1^viii^	59.01 (2)	Al3—Cr1—Al1^xx^	141.98 (4)
Cr1^vi^—Al1—Al1^viii^	107.55 (2)	Al2^xii^—Cr1—Al1^xx^	111.094 (17)
Al1^vii^—Al1—Al1^viii^	60.0	Al2—Cr1—Al1^xx^	111.092 (17)
Cr1^iv^—Al2—Cr1	150.72 (6)	Si1^xvi^—Cr1—Al1^xx^	56.91 (2)
Cr1^iv^—Al2—Al1^xvi^	142.35 (3)	Al1^ix^—Cr1—Al1^xx^	105.74 (3)
Cr1—Al2—Al1^xvi^	60.10 (2)	Al1^xvi^—Cr1—Al1^xx^	105.74 (3)
Cr1^iv^—Al2—Al1^iii^	60.10 (2)	Al2^ii^—Cr1—Al1^xx^	58.77 (2)
Cr1—Al2—Al1^iii^	142.35 (3)	Al2^xv^—Cr1—Al1^xx^	58.77 (2)
Al1^xvi^—Al2—Al1^iii^	109.09 (5)	Al3—Cr1—Cr1^x^	127.136 (8)
Cr1^iv^—Al2—Cr1^ix^	63.51 (2)	Al2^xii^—Cr1—Cr1^x^	163.97 (3)
Cr1—Al2—Cr1^ix^	137.33 (3)	Al2—Cr1—Cr1^x^	60.09 (2)
Al1^xvi^—Al2—Cr1^ix^	79.57 (3)	Si1^xvi^—Cr1—Cr1^x^	57.649 (5)
Al1^iii^—Al2—Cr1^ix^	61.13 (3)	Al1^ix^—Cr1—Cr1^x^	109.35 (2)
Cr1^iv^—Al2—Cr1^x^	137.33 (3)	Al1^xvi^—Cr1—Cr1^x^	60.33 (2)
Cr1—Al2—Cr1^x^	63.51 (2)	Al2^ii^—Cr1—Cr1^x^	56.40 (2)
Al1^xvi^—Al2—Cr1^x^	61.13 (3)	Al2^xv^—Cr1—Cr1^x^	110.49 (4)
Al1^iii^—Al2—Cr1^x^	79.57 (3)	Al1^xx^—Cr1—Cr1^x^	57.03 (2)
Cr1^ix^—Al2—Cr1^x^	110.19 (5)	Al3—Cr1—Cr1^xv^	127.134 (8)
Cr1^iv^—Al2—Al2^ii^	111.077 (13)	Al2^xii^—Cr1—Cr1^xv^	60.09 (2)
Cr1—Al2—Al2^ii^	59.25 (3)	Al2—Cr1—Cr1^xv^	163.97 (3)
Al1^xvi^—Al2—Al2^ii^	105.509 (18)	Si1^xvi^—Cr1—Cr1^xv^	57.652 (5)
Al1^iii^—Al2—Al2^ii^	94.62 (3)	Al1^ix^—Cr1—Cr1^xv^	60.34 (2)
Cr1^ix^—Al2—Al2^ii^	155.09 (5)	Al1^xvi^—Cr1—Cr1^xv^	109.35 (2)
Cr1^x^—Al2—Al2^ii^	55.681 (11)	Al2^ii^—Cr1—Cr1^xv^	110.49 (4)
Cr1^iv^—Al2—Al2^ix^	59.25 (3)	Al2^xv^—Cr1—Cr1^xv^	56.40 (2)
Cr1—Al2—Al2^ix^	111.078 (13)	Al1^xx^—Cr1—Cr1^xv^	57.03 (2)
Al1^xvi^—Al2—Al2^ix^	94.62 (3)	Cr1^x^—Cr1—Cr1^xv^	104.26 (3)
Al1^iii^—Al2—Al2^ix^	105.509 (18)	Al3—Cr1—Al3^xi^	57.49 (4)
Cr1^ix^—Al2—Al2^ix^	55.681 (11)	Al2^xii^—Cr1—Al3^xi^	72.675 (14)
Cr1^x^—Al2—Al2^ix^	155.09 (5)	Al2—Cr1—Al3^xi^	72.673 (14)
Al2^ii^—Al2—Al2^ix^	145.10 (7)	Si1^xvi^—Cr1—Al3^xi^	103.62 (3)
Cr1^iv^—Al2—Al3	97.30 (4)	Al1^ix^—Cr1—Al3^xi^	58.33 (3)
Cr1—Al2—Al3	55.32 (3)	Al1^xvi^—Cr1—Al3^xi^	58.33 (3)
Al1^xvi^—Al2—Al3	96.58 (3)	Al2^ii^—Cr1—Al3^xi^	134.48 (3)
Al1^iii^—Al2—Al3	154.10 (4)	Al2^xv^—Cr1—Al3^xi^	134.48 (3)
Cr1^ix^—Al2—Al3	122.77 (3)	Al1^xx^—Cr1—Al3^xi^	160.53 (4)
Cr1^x^—Al2—Al3	117.33 (3)	Cr1^x^—Cr1—Al3^xi^	114.73 (3)
Al2^ii^—Al2—Al3	81.39 (5)	Cr1^xv^—Cr1—Al3^xi^	114.74 (3)
Al2^ix^—Al2—Al3	68.01 (3)	Al1^xii^—Cr2—Al1^viii^	180.0
Cr1^iv^—Al2—Al3^iv^	55.32 (3)	Al1^xii^—Cr2—Al1^v^	64.71 (3)
Cr1—Al2—Al3^iv^	97.30 (4)	Al1^viii^—Cr2—Al1^v^	115.29 (3)
Al1^xvi^—Al2—Al3^iv^	154.09 (4)	Al1^xii^—Cr2—Al1^iv^	64.71 (3)
Al1^iii^—Al2—Al3^iv^	96.58 (3)	Al1^viii^—Cr2—Al1^iv^	115.29 (3)
Cr1^ix^—Al2—Al3^iv^	117.33 (3)	Al1^v^—Cr2—Al1^iv^	64.71 (3)
Cr1^x^—Al2—Al3^iv^	122.77 (3)	Al1^xii^—Cr2—Al1^vii^	115.29 (3)
Al2^ii^—Al2—Al3^iv^	68.01 (3)	Al1^viii^—Cr2—Al1^vii^	64.71 (3)
Al2^ix^—Al2—Al3^iv^	81.39 (5)	Al1^v^—Cr2—Al1^vii^	115.29 (3)
Al3—Al2—Al3^iv^	58.09 (5)	Al1^iv^—Cr2—Al1^vii^	180.0
Cr1—Al3—Al3^xi^	66.38 (5)	Al1^xii^—Cr2—Al1	115.29 (3)
Cr1—Al3—Fe2	135.56 (5)	Al1^viii^—Cr2—Al1	64.71 (3)
Al3^xi^—Al3—Fe2	158.07 (8)	Al1^v^—Cr2—Al1	180.0
Cr1—Al3—Cr2	135.56 (5)	Al1^iv^—Cr2—Al1	115.29 (3)
Al3^xi^—Al3—Cr2	158.07 (8)	Al1^vii^—Cr2—Al1	64.71 (3)
Cr1—Al3—Al1^xii^	148.29 (3)	Al1^xii^—Cr2—Al3^v^	118.42 (2)
Al3^xi^—Al3—Al1^xii^	104.10 (6)	Al1^viii^—Cr2—Al3^v^	61.58 (2)
Fe2—Al3—Al1^xii^	57.79 (3)	Al1^v^—Cr2—Al3^v^	118.42 (2)
Cr2—Al3—Al1^xii^	57.79 (3)	Al1^iv^—Cr2—Al3^v^	64.67 (3)
Cr1—Al3—Al1^v^	148.29 (3)	Al1^vii^—Cr2—Al3^v^	115.33 (3)
Al3^xi^—Al3—Al1^v^	104.10 (6)	Al1—Cr2—Al3^v^	61.58 (2)
Cr2—Al3—Al1^v^	57.79 (3)	Al1^xii^—Cr2—Al3^iv^	64.67 (3)
Al1^xii^—Al3—Al1^v^	61.97 (5)	Al1^viii^—Cr2—Al3^iv^	115.33 (3)
Cr1—Al3—Al1^vii^	79.23 (4)	Al1^v^—Cr2—Al3^iv^	118.42 (2)
Al3^xi^—Al3—Al1^vii^	145.60 (8)	Al1^iv^—Cr2—Al3^iv^	118.42 (2)
Fe2—Al3—Al1^vii^	56.33 (3)	Al1^vii^—Cr2—Al3^iv^	61.58 (2)
Cr2—Al3—Al1^vii^	56.33 (3)	Al1—Cr2—Al3^iv^	61.58 (2)
Al1^xii^—Al3—Al1^vii^	105.27 (4)	Al3^v^—Cr2—Al3^iv^	115.213 (18)
Al1^v^—Al3—Al1^vii^	105.27 (4)	Al1^xii^—Cr2—Al3^vii^	115.33 (3)
Cr1—Al3—Al3^iv^	99.80 (4)	Al1^viii^—Cr2—Al3^vii^	64.67 (3)
Al3^xi^—Al3—Al3^iv^	127.604 (15)	Al1^v^—Cr2—Al3^vii^	61.58 (2)
Cr2—Al3—Al3^iv^	57.606 (9)	Al1^iv^—Cr2—Al3^vii^	61.58 (2)
Al1^xii^—Al3—Al3^iv^	60.81 (3)	Al1^vii^—Cr2—Al3^vii^	118.42 (2)
Al1^v^—Al3—Al3^iv^	108.89 (3)	Al1—Cr2—Al3^vii^	118.42 (2)
Al1^vii^—Al3—Al3^iv^	56.68 (4)	Al3^v^—Cr2—Al3^vii^	64.787 (18)
Cr1—Al3—Al3^xii^	99.80 (4)	Al3^iv^—Cr2—Al3^vii^	180.0
Al3^xi^—Al3—Al3^xii^	127.606 (15)	Al1^xii^—Cr2—Al3^viii^	61.58 (2)
Fe2—Al3—Al3^xii^	57.606 (9)	Al1^viii^—Cr2—Al3^viii^	118.42 (2)
Cr2—Al3—Al3^xii^	57.606 (9)	Al1^v^—Cr2—Al3^viii^	115.33 (3)
Al1^xii^—Al3—Al3^xii^	108.89 (3)	Al1^iv^—Cr2—Al3^viii^	61.58 (2)
Al1^v^—Al3—Al3^xii^	60.81 (3)	Al1^vii^—Cr2—Al3^viii^	118.42 (2)
Al1^vii^—Al3—Al3^xii^	56.68 (4)	Al1—Cr2—Al3^viii^	64.67 (3)
Al3^iv^—Al3—Al3^xii^	104.01 (5)	Al3^v^—Cr2—Al3^viii^	64.787 (18)
Cr1—Al3—Cr1^xi^	122.51 (4)	Al3^iv^—Cr2—Al3^viii^	64.787 (18)
Al3^xi^—Al3—Cr1^xi^	56.13 (4)	Al3^vii^—Cr2—Al3^viii^	115.213 (18)
Cr2—Al3—Cr1^xi^	101.93 (4)	Al1^xii^—Fe2—Al1^viii^	180.0
Al1^xii^—Al3—Cr1^xi^	56.85 (3)	Al1^xii^—Fe2—Al1^v^	64.71 (3)
Al1^v^—Al3—Cr1^xi^	56.85 (3)	Al1^viii^—Fe2—Al1^v^	115.29 (3)
Al1^vii^—Al3—Cr1^xi^	158.26 (5)	Al1^xii^—Fe2—Al1^iv^	64.71 (3)
Al3^iv^—Al3—Cr1^xi^	114.04 (5)	Al1^viii^—Fe2—Al1^iv^	115.29 (3)
Al3^xii^—Al3—Cr1^xi^	114.04 (5)	Al1^v^—Fe2—Al1^iv^	64.71 (3)
Cr1—Al3—Al2^xii^	55.79 (2)	Al1^xii^—Fe2—Al1^vii^	115.29 (3)
Al3^xi^—Al3—Al2^xii^	70.99 (3)	Al1^viii^—Fe2—Al1^vii^	64.71 (3)
Fe2—Al3—Al2^xii^	118.56 (3)	Al1^v^—Fe2—Al1^vii^	115.29 (3)
Cr2—Al3—Al2^xii^	118.56 (3)	Al1^iv^—Fe2—Al1^vii^	180.0
Al1^xii^—Al3—Al2^xii^	152.70 (4)	Al1^xii^—Fe2—Al1	115.29 (3)
Al1^v^—Al3—Al2^xii^	92.55 (2)	Al1^viii^—Fe2—Al1	64.71 (3)
Al1^vii^—Al3—Al2^xii^	90.15 (3)	Al1^v^—Fe2—Al1	180.0
Al3^iv^—Al3—Al2^xii^	143.76 (7)	Al1^iv^—Fe2—Al1	115.29 (3)
Al3^xii^—Al3—Al2^xii^	60.95 (2)	Al1^vii^—Fe2—Al1	64.71 (3)
Cr1^xi^—Al3—Al2^xii^	102.11 (3)	Al1^xii^—Fe2—Al3^v^	118.42 (2)
Cr1—Al3—Al2	55.79 (2)	Al1^viii^—Fe2—Al3^v^	61.58 (2)
Al3^xi^—Al3—Al2	70.99 (3)	Al1^v^—Fe2—Al3^v^	118.42 (2)
Fe2—Al3—Al2	118.56 (3)	Al1^iv^—Fe2—Al3^v^	64.67 (3)
Cr2—Al3—Al2	118.56 (3)	Al1^vii^—Fe2—Al3^v^	115.33 (3)
Al1^xii^—Al3—Al2	92.55 (2)	Al1—Fe2—Al3^v^	61.58 (2)
Al1^v^—Al3—Al2	152.70 (4)	Al1^xii^—Fe2—Al3^iv^	64.67 (3)
Al1^vii^—Al3—Al2	90.15 (3)	Al1^viii^—Fe2—Al3^iv^	115.33 (3)
Al3^iv^—Al3—Al2	60.95 (2)	Al1^v^—Fe2—Al3^iv^	118.42 (2)
Al3^xii^—Al3—Al2	143.76 (7)	Al1^iv^—Fe2—Al3^iv^	118.42 (2)
Cr1^xi^—Al3—Al2	102.11 (3)	Al1^vii^—Fe2—Al3^iv^	61.58 (2)
Al2^xii^—Al3—Al2	110.12 (4)	Al1—Fe2—Al3^iv^	61.58 (2)
Al1^xvii^—Si1—Al1^vii^	180.000 (16)	Al3^v^—Fe2—Al3^iv^	115.213 (18)
Al1^xvii^—Si1—Al1^xviii^	65.63 (3)	Al1^xii^—Fe2—Al3^vii^	115.33 (3)
Al1^vii^—Si1—Al1^xviii^	114.37 (3)	Al1^viii^—Fe2—Al3^vii^	64.67 (3)
Al1^xvii^—Si1—Al1^xix^	65.63 (3)	Al1^v^—Fe2—Al3^vii^	61.58 (2)
Al1^vii^—Si1—Al1^xix^	114.37 (3)	Al1^iv^—Fe2—Al3^vii^	61.58 (2)
Al1^xviii^—Si1—Al1^xix^	65.63 (3)	Al1^vii^—Fe2—Al3^vii^	118.42 (2)
Al1^xvii^—Si1—Al1^viii^	114.37 (3)	Al1—Fe2—Al3^vii^	118.42 (2)
Al1^vii^—Si1—Al1^viii^	65.63 (3)	Al3^v^—Fe2—Al3^vii^	64.787 (18)
Al1^xviii^—Si1—Al1^viii^	114.37 (3)	Al3^iv^—Fe2—Al3^vii^	180.0
Al1^xix^—Si1—Al1^viii^	180.00 (4)	Al1^xii^—Fe2—Al3^viii^	61.58 (2)
Al1^xvii^—Si1—Al1	114.37 (3)	Al1^viii^—Fe2—Al3^viii^	118.42 (2)
Al1^vii^—Si1—Al1	65.63 (3)	Al1^v^—Fe2—Al3^viii^	115.33 (3)
Al1^xviii^—Si1—Al1	180.0	Al1^iv^—Fe2—Al3^viii^	61.58 (2)
Al1^xix^—Si1—Al1	114.37 (3)	Al1^vii^—Fe2—Al3^viii^	118.42 (2)
Al1^viii^—Si1—Al1	65.63 (3)	Al1—Fe2—Al3^viii^	64.67 (3)
Al1^xvii^—Si1—Cr1^xiii^	61.518 (11)	Al3^v^—Fe2—Al3^viii^	64.787 (18)
Al1^vii^—Si1—Cr1^xiii^	118.482 (11)	Al3^iv^—Fe2—Al3^viii^	64.787 (18)
Al1^xviii^—Si1—Cr1^xiii^	61.519 (11)	Al3^vii^—Fe2—Al3^viii^	115.213 (18)

Symmetry codes: (i) *x*−2/3, *y*−1/3, *z*−1/3; (ii) −*x*+*y*+1/3, −*x*+2/3, *z*−1/3; (iii) −*x*+1/3, −*y*+2/3, −*z*+2/3; (iv) *x*−*y*, *x*, −*z*+1; (v) −*x*, −*y*, −*z*+1; (vi) *y*−1/3, −*x*+*y*+1/3, −*z*+1/3; (vii) −*x*+*y*, −*x*, *z*; (viii) −*y*, *x*−*y*, *z*; (ix) −*y*+2/3, *x*−*y*+1/3, *z*+1/3; (x) *y*+1/3, −*x*+*y*+2/3, −*z*+2/3; (xi) −*x*+2/3, −*y*+1/3, −*z*+4/3; (xii) *y*, −*x*+*y*, −*z*+1; (xiii) *x*−*y*−1/3, *x*−2/3, −*z*+1/3; (xiv) −*x*+2/3, −*y*+1/3, −*z*+1/3; (xv) *x*−*y*+1/3, *x*−1/3, −*z*+2/3; (xvi) *x*+2/3, *y*+1/3, *z*+1/3; (xvii) *x*−*y*, *x*, −*z*; (xviii) −*x*, −*y*, −*z*; (xix) *y*, −*x*+*y*, −*z*; (xx) *x*−*y*+2/3, *x*+1/3, −*z*+1/3.

## References

[bb1] Brandenburg, K. & Putz, H. (2017). *DIAMOND*. Crystal Impact GbR, Bonn, Germany.

[bb2] Bruker (2023). *APEX5* and *SAINT*. Bruker AXS Inc. Madison, Wisconsin, USA, 2008.

[bb3] Hu, Q., Wen, B. & Fan, C. (2021). *IUCrData* **6**, x210988. 10.1107/S2414314621009883 PMC9462374 36338944

[bb4] Ko, H., Gourdon, O., Gout, D., Mun, E.-D., Thimmaiah, S. & Miller, G. J. (2010). *Inorg. Chem.* **49**, 11505–11515. 10.1021/ic101671k 21077651

[bb5] Krause, L., Herbst-Irmer, R., Sheldrick, G. M. & Stalke, D. (2015). *J. Appl. Cryst.* **48**, 3–10. 10.1107/S1600576714022985 PMC4453166 26089746

[bb6] Liu, C. & Fan, C. (2018). *IUCrData* **3**, x180363.

[bb7] Ma, H., He, Z., Hou, L. & Steurer, W. (2018). *J. Alloys Compd.* **765**, 753–756.

[bb8] Ma, H., You, L. & He, Z. (2020). *Mater. Charact.* **166**, 110424.

[bb9] Sheldrick, G. M. (2015*a*). *Acta Cryst.* A**71**, 3–8.

[bb10] Sheldrick, G. M. (2015*b*). *Acta Cryst.* C**71**, 3–8.

[bb11] Westrip, S. P. (2010). *J. Appl. Cryst.* **43**, 920–925.

