# Supplementary material for: Al48.18Cr22.78Fe4.04Si3
Source: IUCrdata. 2025 Nov 21;10(Pt 11):x251039. doi: 10.1107/S2414314625010399 (PMC12810298; doi:10.1107/S2414314625010399)
Supplement: Supplementary file 3 [file x-10-x251039-sup3.zip › esi/supp.docx]

**SUPPLEMENTARY MATERIALS:**

**Crystal structure of Al_48.18_Cr_22.78_Fe_4.04_Si_3_**

**Xinyu Liang**^a^, **Changzeng Fan**^a,b,^***, Bin Wen**^a^ **and Lifeng Zhang**^c^

^a^ State Key Laboratory of Metastable Materials Science and Technology, Yanshan University,

Qinhuangdao 066004, People’s Republic of China

^b^ Hebei Key Lab for Optimizing Metal Product Technology and Performance, Yanshan University, Qinhuangdao, Hebei 066004, People’s Republic of China

^c^ School of Mechanical and Materials Engineering, North China University of Technology, Beijing, People’s Republic of China

*Correspondence email: [chzfan@ysu.edu.cn](mailto:chzfan@ysu.edu.cn)

The chemical compositions were examined quantitatively by energy dispersive X-ray spectroscopy (EDX) analysis attached to a Hitachi S-3400N SEM for the purpose of guiding the crystal structure refinement. The examined points and areas are designated in Fig. S1, and the corresponding results are listed in Table S1. The deviation relative to the results of refinement of chemical composition is probably caused by the tilt of the single crystal surface to the incident beam. In addition, the conductive adhesives and glues may also result in the detected impurity elements of carbon. For ease of reading, the atomic ratio of Al, Cr, Fe, Si and C was calculated and shown in the last column of Table S1.


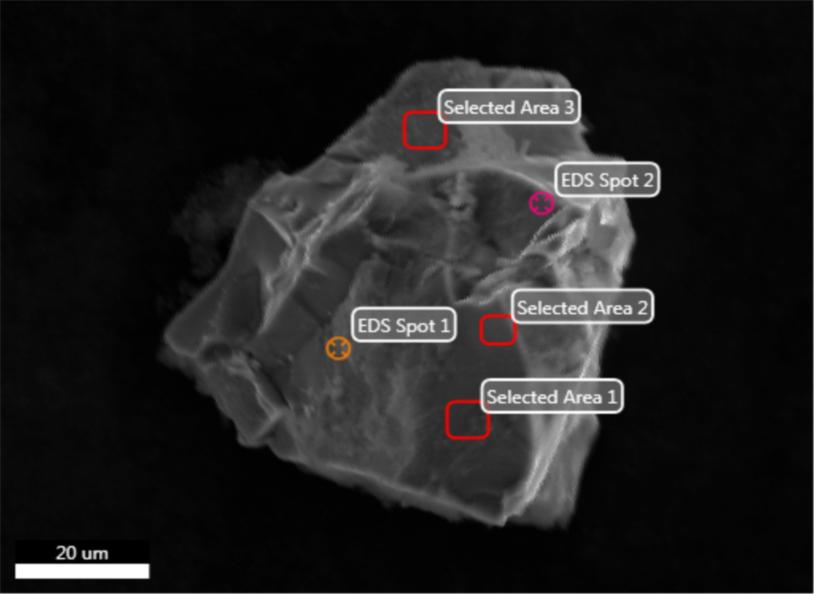


Fig. S1 Single crystal of Al_48.18_Cr_22.78_Fe_4.04_Si_3_ with selected spots and areas for EDX analysis

**Table S1 EDX results for selected points and areas as designated in Fig. S1**

|  | Element | Weight (%) | Atomic (%) | Error (%) | Al:Cr:Fe:Si |
| --- | --- | --- | --- | --- | --- |
| Spot1 | C | 40.36 | 68.70 | 9.64 | 47.4: 38.1: 8.3: 6.3 |
|  | Al | 19.55 | 14.82 | 5.23 |  |
|  | Si | 2.71 | 1.97 | 6.58 |  |
|  | Cr | 30.29 | 11.91 | 2.04 |  |
|  | Fe | 7.10 | 2.60 | 3.95 |  |
| Spot2 | C | 20.39 | 43.28 | 11.19 | 57.4: 26.5: 7.2: 9.0 |
|  | Al | 34.42 | 32.53 | 5.08 |  |
|  | Si | 5.61 | 5.09 | 7.05 |  |
|  | Cr | 30.69 | 15.05 | 2.03 |  |
|  | Fe | 8.89 | 4.06 | 3.53 |  |
| Area1 | Al | 38.40 | 52.56 | 5.85 | 52.6: 28.2: 9.7: 9.6 |
|  | Si | 7.31 | 9.61 | 7.61 |  |
|  | Cr | 39.64 | 28.15 | 2.16 |  |
|  | Fe | 14.65 | 9.69 | 3.60 |  |
| Area2 | C | 26.38 | 52.32 | 10.62 | 55.4: 28.9: 8.6: 7.1 |
|  | Al | 29.93 | 26.43 | 5.20 |  |
|  | Si | 3.96 | 3.36 | 7.08 |  |
|  | Cr | 30.09 | 13.79 | 2.02 |  |
|  | Fe | 9.63 | 4.11 | 3.47 |  |
| Area3 | C | 20.30 | 43.02 | 11.03 | 57.9: 27.2: 6.0: 8.9 |
|  | Al | 34.95 | 32.98 | 5.02 |  |
|  | Si | 5.61 | 5.09 | 7.02 |  |
|  | Cr | 31.61 | 15.48 | 2.00 |  |
|  | Fe | 7.53 | 3.43 | 3.80 |  |

The Al, Cr, Fe and Si atoms were refined against different positions as shown in Table S2. From the results it is derived that the reported refinement (in bold) is the most suitable one that fit the EDX results.

| Position | | | | | | Compositions | | | | R_1_ | | |
| --- | --- | --- | --- | --- | --- | --- | --- | --- | --- | --- | --- | --- |
| *3a* | *3b* | *18f* | *18h* | *18h* | *18h* | Al | Cr | Fe | Si |  | | |
| Cr | Al | Al | Si/Fe | Cr | Al | 39 | 21 | 4.58 | 13.42 | 3.23 | | |
| Cr | Al | Al/Fe | Si(NPD) | Cr | Al | 43.46 | 21 | -4.4 | 18 | 10.33 | | |
| Cr | Al/Fe | Al | Si(NPD) | Cr | Al | 38.97 | 21 | 0.03 | 18 | 5.61 | | |
| Cr | Al | Al | Si(NPD) | Cr | Al/Fe | 54.85 | 21 | -15.8 | 18 | 23.88 | | |
| Cr/Fe | Al | Al | Si | Cr | Al | refinement unstable | | | | | | |
| Cr | Al | Al | Si | Cr/Fe | Al | refinement unstable | | | | | | |
| Cr | Si | Al | Al/Cr/Fe | Cr | Al | 48.50 | 22.91 | 3.59 | 3 | | 3.22 | |
| Cr | Si | Al | Al/Cr/Fe | Cr | Si | 30.26 | 23.15 | 3.58 | 21 | | 3.40 | |
| Cr | Al | Al | Al/Cr/Fe | Cr | Al | 51.66 | 22.21 | 4.13 | 0 | | 3.24 | |
| Cr | Al | Al | Al/Cr/Fe | Cr | Si | 33.35 | 22.85 | 3.80 | 18 | | 3.41 | |
| Cr | Al | Si | Al/Cr/Fe | Cr | Si | 15.23 | 23.04 | 3.73 | 36 | | 3.50 | |
| Cr | Al | Si | Al/Cr/Fe | Cr | Al | 33.57 | 22.21 | 4.21 | 18 | | 3.39 | |
| Cr | Si | Si | Al/Cr/Fe | Cr | Al | 30.49 | 22.47 | 4.04 | 21 | | 3.38 | |
| Cr | Si | Si | Al/Cr/Fe | Cr | Si | 12.15 | 23.37 | 3.49 | 39 | | 3.50 | |
| Cr | Si | Al | Al/Cr/Fe | Fe | Al | Negative occupancy for Cr(18h) | | | | | | |
| Cr | Si/Al | Al | Al/Cr/Fe | Cr | Al | Negative occupancy for Al(3b) | | | | | | |
| Cr | Si | Al/Si | Al/Cr/Fe | Cr | Al | refinement unstable | | | | | | |
| Cr | Si | Al | Al/Cr/Fe | Cr | Al/Si | refinement unstable | | | | | | |
| **Cr/Fe** | **Si** | **Al** | **Al/Cr/Fe** | **Cr** | **Al** | **48.18** | **22.78** | **4.04** | **3** | | | **3.07** |
| Fe | Si | Al | Al/Cr/Fe | Cr | Al | 48.17 | 19.94 | 6.89 | 3 | | | 3.23 |
| Fe | Si | Al | Al/Cr/Fe | Cr | Si | 29.89 | 20.49 | 6.61 | 21 | | | 3.31 |
| Fe | Al | Al | Al/Cr/Fe | Cr | Al | 51.13 | 20.39 | 6.47 | 0 | | | 3.25 |
| Fe | Al | Al | Al/Cr/Fe | Cr | Si | 32.96 | 20.30 | 6.74 | 18 | | | 3.32 |
| Fe | Al | Si | Al/Cr/Fe | Cr | Si | 14.69 | 21.34 | 5.97 | 36 | | | 3.42 |
| Fe | Al | Si | Al/Cr/Fe | Cr | Al | 33 | 20.61 | 6.39 | 18 | | | 3.40 |
| Fe | Si | Si | Al/Cr/Fe | Cr | Al | 29.92 | 20.79 | 6.29 | 21 | | | 3.36 |
| Fe | Si | Si | Al/Cr/Fe | Cr | Si | 11.52 | 20.11 | 5.37 | 39 | | | 3.40 |
| Fe | Si | Al | Al/Cr/Fe | Fe | Al | Negative occupancy for Cr(18h) | | | | | | |

**Table S2 Different choices of refinement and the resulting refined chemical compositions**
