# Supplementary figures and images for: Al48.18Cr22.78Fe4.04Si3
Source: IUCrdata. 2025 Nov 21;10(Pt 11):x251039. doi: 10.1107/S2414314625010399 (PMC12810298; doi:10.1107/S2414314625010399)

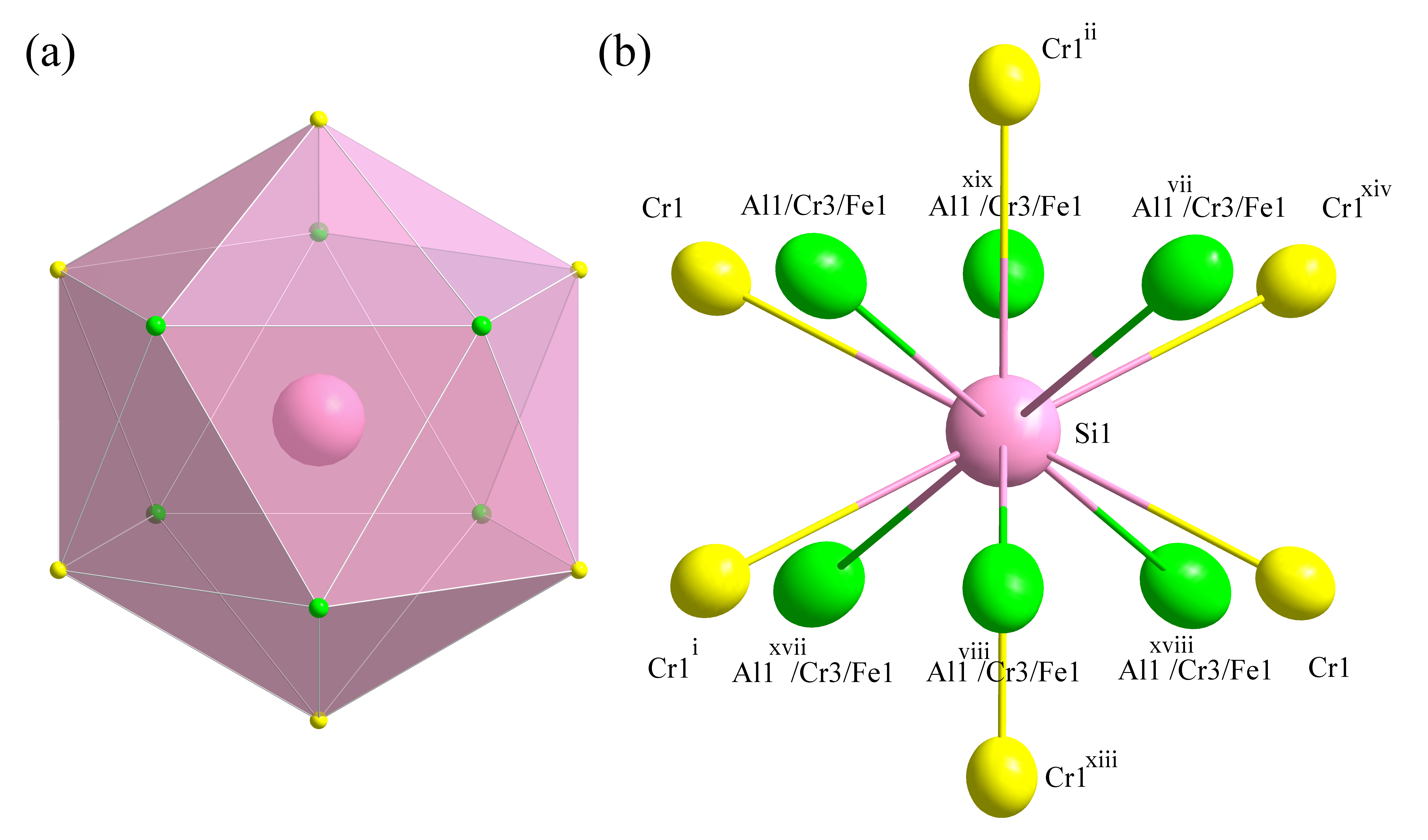

Supplement: Supplementary file 3 [file x-10-x251039-sup3.zip › esi/Figure 2.tif]
